# Prevalence and determinants of depression among patients with Type 2 diabetes mellitus attending family medicine clinics in Qatar

**DOI:** 10.1016/j.ajmo.2022.100014

**Published:** 2022-05-29

**Authors:** Mansoura Ismail, Mai Hassan Seif, Nourhan Metwally, Marwa Neshnash, Anwar I. Joudeh, Muna Alsaadi, Samya Al-Abdulla, Nagah Selim

**Affiliations:** 1 Primary Health Care Corporation, Doha-Qatar; 2 Family Medicine Department, Faculty of Medicine, Suez Canal University, Ismailia, Egypt; 3 Internal Medicine Department, Al-Khor Hospital, Hamad Medical Corporation, Doha-Qatar; 4 Internal Medicine Department, Faculty of Medicine, University of Jordan, Amman, Jordan; 5 Public Health Department, Faculty of Medicine, Cairo University, Cairo, Egypt

**Keywords:** Depression, Diabetes Mellitus, Glycemic Control, Primary care

## Abstract

**Aims:**

To assess the prevalence of depression and its associated factors among patients with Type 2 diabetes mellitus attending family medicine clinics in Qatar

**Methods:**

A cross-sectional study was conducted from January to April 2021 where 683 adult patients with Type 2 diabetes mellitus were selected by cluster sampling technique using probability-proportionate to size sampling. Diabetes mellitus was defined as having HA1c of greater than or equal to 6.5%, and patients were assessed for depression using the Patient Health Questionnaire-9. The relationship between depression, glycemic control, and background characteristics was analyzed using Chi-square, and binary logistic regression analyses. Adjusted logistic regression models estimated the significant factors that were independently associated with depression.

**Results:**

20.1% of the study participants had depression with the vast majority of them having mild depression (70.8%). More than three-quarters had uncontrolled diabetes mellitus (81.5%). Male patients were at higher risk for developing depression (AOR =1.98, 1.25-3.14) when compared to female patients. On the other hand, being Qatari was associated with a lower risk for depression compared to non-Qatari patients (AOR =0.56, 0.34—0.90), and treatment with insulin-containing regimens was associated with a lower risk for depression as compared to treatment with non-insulin- containing regimens (AOR =0.49, 0.30-0.78).

**Conclusions:**

Prevalence of depression among patients with Type 2 diabetes attending family medicine clinics in Qatar is high. Therefore, utilizing a multidisciplinary health care plan for screening and management of depression in patients with diabetes in a primary health care setting is highly recommended.

**Funding:**

The authors have not declared a specific grant for this research from any funding agency in the public, commercial or not-for-profit sectors.

## 1 Introduction

Diabetes mellitus (DM) is a common chronic non-communicable disease that is considered a leading cause for disability and mortality worldwide with around 12% of healthcare expenses in many countries dedicated to preventing and combating diabetes complications^1^. In addition to its associated health and economic burden, DM imposes social and psychological challenges that would ultimately increase the risk for depression.^2^ According to the WHO, 265 million people worldwide have depression.^3^ Comorbid depression complicates the management of chronic diseases and it is associated with worse clinical outcomes compared to having depression alone, having any of the chronic diseases alone, or having a combination of other chronic diseases other than depression.^4^ Depression in patients with diabetes is often unrecognized and untreated leading to poor adherence to diabetes self-care plans and resulting in potentially negative health consequences including a higher risk for diabetic complications, functional impairment, and increased healthcare expenditure.^5^^,^^6^

Depression is the second-leading cause of disability worldwide with a higher likelihood of occurrence in patients with diabetes.^7^ Katon estimated that 15%-20% of people with diabetes are struggling with depression, more likely having moderate to severe form of depression. He also hypothesized that the relationship between diabetes and depression is bidirectional with one disease leading to the increased risk of having the other.^5^ The etiology of depression in diabetes is not known yet but it is probably due to the complex interaction of genetic, biological, and psychological factors. Vascular changes due to DM could be the biological basis for the development of depression among patients with diabetes. Several neurotransmitters and neuron-endocrine defects have been identified to be common for both depression and diabetes, adding to the etiological speculations.^8^

Even as separate entities, diabetes and depression are by themselves major health problems in the world. The majority of studies on the burden of depression in diabetes mellitus (DM) have been carried out in high-income countries but little is known about this in developing countries.^9^, ^10^, ^11^ A worldwide survey conducted through 60 countries using the ICD-10 criteria found that the one-year prevalence of depressive episodes in people with diabetes was 9.3% as compared to 3.2% in people without diabetes.^4^ An earlier matched case-control study on adults attending primary health care in Qatar found that depression scores were significantly higher and more frequent in patients with diabetes in comparison to healthy controls. Using the short version of the Depression Anxiety Stress Scales (DASS)-21, 38.9% of diabetic patients had mild depression compared to 26.5% of controls (p<0.001), and 13.6% of diabetic patients had severe depression compared to 5.9% of controls (p<0.001).^10^ The high prevalence of depression among patients with diabetes had led to the term "diapression".^12^

It is likely that the complex interactions between diabetes and social and cultural dynamics affect the way people experience illness and health.^13^ As noted from previous studies, the prevalence of comorbid depression in diabetes and its associated potential risk factors have been variably reported in different studies, populations and treatment settings.^14^, ^15^, ^16^, ^17^, ^18^, ^19^, ^20^, ^21^, ^22^, ^23^ Hence, it is important to take a culture-centered approach to assess and address risk factors for developing depression in patients with diabetes.

Primary healthcare is the front door of healthcare services for the community. Therefore, it should participate actively in screening, case finding, and management of depression among people with diabetes. To date, there is a lack of data on the prevalence of depression and its associated sociodemographic and clinical features in patients with diabetes treated in primary health care clinics in Qatar. This study aimed to assess this prevalence and its associated factors among patients with Type 2 diabetes mellitus attending family medicine clinics in Qatar. This will help in planning structured and targeted patient care services in order to enhance metabolic control, improve the clinical outcomes and possibly reduce the health-related expenses of patients with diabetes. Due to the unique sociodemographic structure of Qatar population, we hypothesize that the prevalence and associated factors of depression among patients with diabetes could be different from studies done in other countries.

## 2 Methodology

A cross-sectional study was conducted from January to April 2021. All adult patients (age ≥ 18), with Type 2 DM (defined as having a HA1c of greater than or equal to 6.5%) who were attending family medicine clinics at primary healthcare corporation (PHCC) in Qatar were invited to participate in this study. Patients who were younger than 18 years old, had type 1 DM or gestational diabetes, had a previous diagnosis of depression or other psychiatric disorder, or were not capable of independent communication were excluded from the study.

In Qatar, there are 27 publicly run health centers providing healthcare for patients with diabetes through family medicine clinics which are both appointment-based and walk-in clinics. Sample size calculation was based on the hypothesis that the prevalence of depression among patients with type 2 diabetes mellitus is 50%^23^ +/- 5% to get the maximum sample size with a 5% margin of error, and 95% level of confidence. The design effect for cluster sampling was 1.5. The sample size was 638, and cluster sampling technique was used in which PHCC centers were considered the primary sampling units. The clusters were identified based on the number of registered diabetic patients in each health center. A simple random sample was conducted to select four health centers, and within each selected health center, all eligible patients were included in the study. The estimated sample size was distributed proportionately among the four selected health centers according to the size of the registered patients with Type 2 DM (probability-proportionate to size sampling).

## 3 Data collection

A face-to-face interview was conducted by well-trained nurses using a questionnaire that included: socio-demographic characteristics including age (≤60 years or >60 years), gender (male or female), marital status (married or single), smoking (non-smoker, smoker, ex-smoker), family history of diabetes (yes or no), as well as health factors including diabetes control (HA1c >7% or ≤ 7%), treatment modalities (lifestyle modifications only, insulin-containing regimens or use of oral hypoglycemic agents only), duration of diabetes (<5 years, 5-10 years or ≥10 years), and the presence of other co-morbidities (hypertension, dyslipidemia, hypothyroidism, obesity). Physical examination data within the last three months were obtained from the patient's medical record including BMI, blood pressure, fundoscopy and HbA1c. The instrument was adopted and translated to the Arabic language, and back to English to be tested for validity. Depression status of patients was ascertained at the time of recruitment by using the Patient Health Questionnaire 9 (PHQ 9) which is a 9-item questionnaire designed to correspond to the nine diagnostic criteria for major depressive disorder covered in the Diagnostic and Statistical Manual of Mental Disorders (DSM–IV). In this questionnaire, a total score of 0 indicates no depression, 1–4 indicates minimal depression; 5–9 mild depression; 10–14 indicates moderate depression, a score of 15–19 signifies moderately severe depression, and a score 20-27 signifies severe depression.^24^ This questionnaire has been validated for use in primary care,^25^ and it showed good internal (Cronbach's alpha=0.85) and test re-test reliability (intraclass correlation coefficient=0.92).^26^

In this study, a PHQ-9 score of 4 to 9 was used to define mild depression, 10-19 for moderate depression and more than 20 for severe depression. Glycemic status was categorized as a good glycemic control if HbA1c was equal to or less than 7% or poor glycemic control if HbA1c >7%. Blood pressure less than 130/80, LDL less than 100 mg/dl and BMI less than 25 were considered normal, whereas BMI of 25 to <30 is considered overweight and BMI of 30 or higher is considered obesity.

**Data processing and analyses**

Data were analyzed using SPSS version 20. The analysis was performed and presented as tables and figures that included frequency and percentages for categorical variables. Bivariate analysis using Chi-square test (X^2^) was used to assess association (crude odds ratio) between the categorical dependent variable (depression) and independent variables (socio-demographic, clinical characteristics including BMI and HbA1c and smoking history). Binary logistic regression was performed to identify the most significant associated factors of the outcome variable (depression). We used enter method model in the regression analysis and selected the significant variables from bivariante analysis. An alpha (p) value of ≤ 0.05 was considered the cut-off level for statistically significant association.

### 3.1 Ethical considerations

Ethical approval was obtained from the Institutional Review Board (IRB) of the research department in primary care corporation in Qatar. Informed verbal consent was obtained from each study participant, where they were informed about the aim of the study and its nature and their right to interrupt the interview at any time. Patients’ confidentiality and privacy were preserved at all levels of the study. Patients with diabetes who were diagnosed with depression during the study were managed accordingly.

## 4 Results

**Figure 1:**
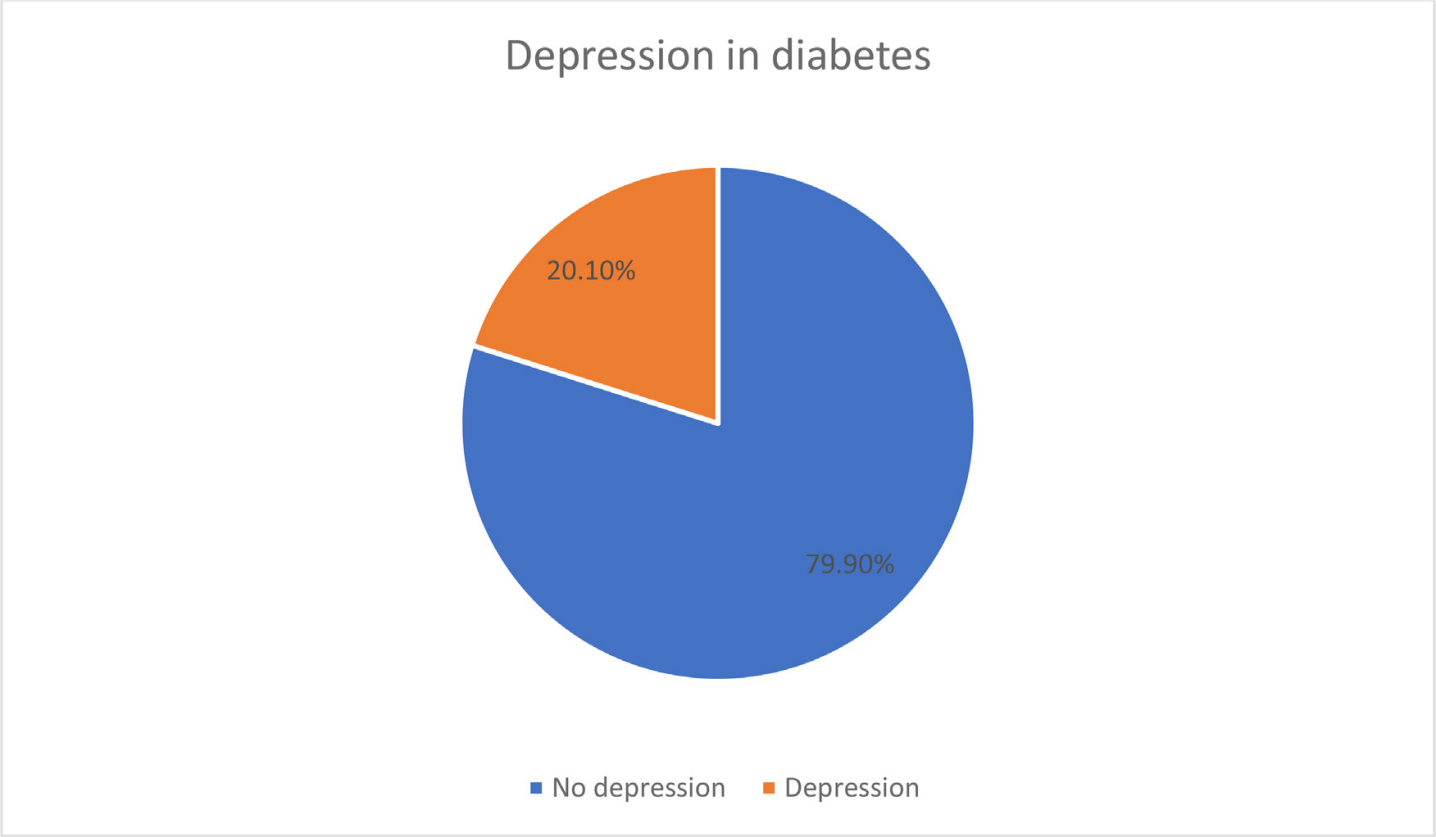
Prevalence of depression among type 2 diabetic patients attending family medicine clinics in Qatar.

Six hundred and eighty-three patients with Type 2 DM were enrolled in the current study. The majority were married (92.2%), two-thirds were males (61.1%), and almost half of them were Qatari and aged less than 50 years (48.8% and 48.2% respectively). Approximately three-quarters of the studied subjects had a family history of diabetes (72.0%). More than two-thirds of the patients were on oral hypoglycemic medications (69.7%) and 67.2% of the total patients developed diabetic complications (Table 1). The prevalence of depression among patients with Type 2 DM was 20.1 %, with the vast majority of them having mild depression (70.8%), while 4.7 % and 1.2% had moderate and severe depression respectively (Figure 1). The association between various sociodemographic characteristics and depression showed that depression was found to be statistically significantly higher among patients who were females (p <0.001), married (p = 0.018), Qatari (p <0.001), their age more than 50 years (p = 0.013),and with family history of diabetes (p = 0.002) (Table 2). However, the clinical characteristics of patients including blood pressure control, lipid panel level, TSH, and vitamin D status were not statistically significantly correlated with the prevalence of depression in diabetes (Table 3).

**Table 1:** Sociodemographic & clinical characteristics of the studied group.

		No	%
Nationality	Others	350	51.2%
	Qatari	333	48.8%
Gender	Female	266	38.9%
	Male	417	61.1%
Age Group	Lower than or equal 60 years	329	48.2%
	More than 60 years	354	51.8%
Marital Status	Married	630	92.2%
	Single, widow, divorced	53	7.8%
Family History of Diabetes	No	191	28.0%
	Yes	492	72.0%
Smoking	Non-smoker	515	75.4%
	Smoker	163	23.9%
	Ex-smoker	5	0.7%
Treatment for Diabetes	Lifestyle only	19	2.8%
	Oral Hypoglycemic Agents only	467	69.7%
	Oral Hypoglycemic Agents& insulin	156	27.5%
Diabetic complications	Free	224	32.8%
	Have complications	593	67.2%

**Table 2:** Relationship of depression with sociodemographic characteristics, diabetes duration and control among patients with Type 2 diabetes mellitus at primary health care in Qatar.

			Depression Score				Chi square	P value
			Free		Depression			
			No	%	No	%		

GENDER	Female		189	34.6%	77	56.2%	21.47	<0.001
	Male		357	65.4%	60	43.8%		
Marital Status	Married		497	91.0%	133	97.1%	5.61	0.018
	Single		49	9.0%	4	2.9%		
Nationality	Others		305	55.9%	45	32.8%	23.22	0.000
	Qatari		241	44.1%	92	67.2%		
Age Group	Lower than or equal 60 years		276	50.5%	53	38.7%	6.17	0.013
	More than 60 years		270	49.5%	84	61.3%		
Family history of Diabetes	No		167	30.6%	24	17.5%	9.28	0.002
	Yes		379	69.4%	113	82.5%		
Smoking	Non-smoker		396	72.5%	119	86.9%	12.29	0.002
	Smoker		108	19.8%	14	10.2%		
	X-smoker		42	7.7%	4	2.9%		
Hb1Ac	High		414	81.2%	110	82.7%	0.16	0.686
	Low		96	18.8%	23	17.3%		
Duration of diabetes	Less than 5 years		134	87.0%	20	13.0%	24.29	<0.001
	From 5-10 years		160	82.1%	35	17.9%		
	More than 10 years		160	67.4%	78	32.6%		

**statistically significant at p 0.05

**Table 3:** Relationship of depression with clinical characteristics among patients with Type 2 diabetes mellitus at primary health care in Qatar.

		Depression Score								Chi square	P value
		Free		Mild Depression		Moderate Depression		Severe Depression			
		No	%	No	%	No	%	No	%		

Blood pressure	Normal Blood pressure	216	50.5%	50	61.0%	10	55.6%	3	60.0%	3.25	0.355
	Hypertensive	212	49.5%	32	39.0%	8	44.4%	2	40.0%		
LDL	Normal	395	72.3%	76	78.4%	17	53.1%	6	75.0%	7.68	0.053
	Abnormal	151	27.7%	21	21.6%	15	46.9%	2	25.0%		
Vit D	Normal	69	12.6%	16	16.5%	2	6.3%	0	0.0%	3.62	0.306
	Abnormal	477	87.4%	81	83.5%	30	93.8%	8	100.0%		
Cholesterol	Normal	381	69.8%	74	76.3%	17	53.1%	6	75.0%	6.27	0.099
	Abnormal	165	30.2%	23	23.7%	15	46.9%	2	25.0%		
TSH	Normal	399	73.1%	71	73.2%	22	68.8%	6	75.0%	0.31	0.958
	Abnormal	147	26.9%	26	26.8%	10	31.3%	2	25.0%		
Triglycerides	Normal	302	55.3%	59	60.8%	20	62.5%	5	62.5%	1.64	0.651
	Abnormal	244	44.7%	38	39.2%	12	37.5%	3	37.5%		
BMI	Normal or underweight	93	17.0%	21	21.6%	0	0.0%	1	12.5%	8.21	0.042
	Obese	453	83.0%	76	78.4%	32	100.0%	7	87.5%		
Hb1Ac	High	414	81.2%	76	80.0%	26	86.7%	8	100.0%		
	Normal	96	18.8%	19	20.0%	4	13.3%	0	0.0%	..	..

*Statistically significant at p<0.05

Binary logistic regression analysis for depression (as a dependent variable) with multiple independent variables including gender, nationality, pharmacological treatment for diabetes and duration of diabetes mellitus showed that males were at a higher risk of depression (AOR= 1.98, 95% CI 1.25-3.15, *p* = 0.004), while being Qatari and treatment for diabetes with insulin were at lower risk for depression development (AOR = 0.56, 95% CI 0.34-0.90, *p* = 0.017, 0.49, 95% CI 0.30-0.78 *p* =0.003 respectively). Moreover, duration of diabetes for more than 10 years was also associated with a higher risk for having depression as shown in Table 4 (AOR = 3.25, 95% CI 1.89- 5.58 *p* <0.001).

**Table 4:** Binary logistic regression analysis for depression (dependent variable) with multiple independent variables including gender, nationality, pharmacological treatment for diabetes and duration of diabetes mellitus.

		Wald	P value	OR	95% C.I .for OR	
					Lower	Upper
	GENDER(Male)	8.48	0.004	1.98	1.251	3.146
	Nationality (Qatari)	5.65	0.017	0.56	0.341	0.902
	Treatment for Diabetes	10.18	0.006			
	Oral Hypoglycemic Agents	2.58	0.109	0.18	0.023	1.458
	Insulin-containing regimens	8.85	0.003	0.49	0.302	0.782
	Duration of Diabetes (5-10 years)	1.58	0.208	1.47	0.808	2.658
	Duration of Diabetes (>10 years)	18.12	<0.001	3.25	1.888	5.582
	Constant	13.98	<0.001	0.40		

## 5 Discussion

The current study aimed to assess the prevalence and associated factors of depression among patients with Type 2 DM attending family medicine clinics in PHCC, Qatar. The prevalence of depression in this study with a PHQ-9 cutoff value of more than 4 was (20.1%) where most of the cases had mild depression (70.8%).

Worldwide, the prevalence of depression among patients with Type 2 DM varied widely in different countries and studies. The reported prevalence of depression in Type 2 DM was 10.6% in Taiwan,^14^ 11.5% in Malaysia,^15^ 13% in Addis Ababa,^16^ 14.7% in Pakistan,^17^ 18.6% in Brazil,^18^ 19.7% in Jordan,^19^ 23.2% in Vietnam,^20^ 38.8% in India,^21^ 40% in Palestine,^22^ and 48% in Saudi Arabia.^23^ The criteria used to determine depression in these studies varied according to different instruments used such as The Beck Depression Inventory (BDI), PHQ-9, Center for Epidemiologic Studies Depression Scale (CESD), Hospital Anxiety and Depression Scale (HADS) and the Depression, Anxiety and Stress Scale - 21 Items (DASS-21).^27^. Thus, the difference in the prevalence rate of co-morbid depression in patients with Type 2 DM among different studies could be explained by using different assessment tools for depression, sociocultural, and behavioral-related factors among study participants as well as using different cutoff scores for diagnosing depression even with the same instrument.

The study findings indicated a higher prevalence of depression in patients with Type 2 DM compared to another recent study in Qatar showing a prevalence of 15.4%.^28^ This could be explained by the fact that the earlier study included only Qatari adults who were younger than the age of 60 years, whereas in the current study only half of the participants had Qatari nationality and just more than half were aged more than 60 years.

In the current study, male gender was associated with developing depression, which is in line with study results from Ethiopia, where male patients had a 1.92 higher chance of developing comorbid depression in diabetes in comparison to female patients.^29^ However, other studies both locally and internationally showed that female patients with Type 2 DM had a higher incidence of depression.^22^^,^^23^^,^^28^ Another cross-sectional study in Austria also found that women with Type 2 diabetes were twice as likely to be diagnosed with depression compared with men.^30^ However, a recent review by Lloyd et al. concluded that studies from around the world confirm that the prevalence of depression is increased in people with diabetes mellitus although the levels of depression vary between countries, between populations within the same country and between the sexes. Consequently, healthcare providers should manage mental health disorders in patients with diabetes in a culture-centered approach.^13^

Although the current study showed a statistically significant correlation between being married and depression, marital status was not found to be an associated factor for developing depression in patients with Type 2 DM in other studies. Previous studies in Sir Lanka and Ethiopia suggested that the marital status of patients with DM plays a role with those being married having less rates of depression in contrast to those who were single/divorced or widowed.^29^^,^^31^ On the other hand, the result of the study conducted in Vietnam reported that marital status had no significant association with depression in people with diabetes.^20^ The increased prevalence of depression among married patients with diabetes in this study may suggest that lifestyle and social factors among this subgroup of patients could be involved. Notably, Qatar has a very distinctive population with 88% of the 2.9 million residents being expatriates, 75% are males with a population median age of 33 years.^32^ This could be associated with unique social and cultural dynamics affecting response to stress and health.

Regarding treatment with insulin, both our study and the recent study from Qatar^28^ showed that patients with Type 2 DM who were treated with insulin had a lower risk for developing depression than other patients. On the contrary, other studies found a higher prevalence and severity of depression among patients who were on insulin.^33^ One possible explanation for this finding is that patients in our sample who received insulin might have had better control of their diabetes. The interpretation of potential risk factors for depression in diabetes is further complicated by the interaction between these factors. For example, using insulin in Type 2 diabetes might be associated with a longer duration of the disease and a higher risk of diabetes complications; however, patients in our study may not be able to afford insulin or were not receiving insulin for the appropriate reasons. Therefore, further research is needed to explore the underlying etiology behind this apparent association.

The correlation of age with comorbid depression in diabetes has been inconstantly reported. In the current study, depression was statistically significantly associated with patients’ age. In the other study from Qatar, younger patients were more likely to report depressive symptoms.^28^ Studies conducted in Saudi Arabia showed a high prevalence of depression among patients older than 60 years.^23^ On the other hand, the result of the study conducted in Vietnam reported that the prevalence of depression was significantly high among patients under 60 years old.^20^

This study showed an association between obesity and depression among patients with Type 2 DM. Similar findings from previous studies reported such association as depression was documented to be strongly associated with obesity among Middle Eastern societies, including Qatar.^34^

Approximately two-thirds of the studied group had uncontrolled diabetes mellitus with HbA1cmore than 7%. In contrast to other study reports, we did not find a significant association between poor glycemic control and depression. Other studies have reported that depressed patients with Type 2 DM have higher HbA1c levels compared with non-depressed patients.^35^^,^^36^ However, this association has not been verified in all studies. In a systematic review and meta-analyses by Ismail, et al., depression was found to be weakly associated with the glycemic status.^37^

This study has several limitations. As a cross-sectional study, a causal relationship between diabetes and depression cannot be established; therefore, a longitudinal study design is needed to investigate this relationship. In addition, as it was only conducted in primary health care, the study results might not reflect the actual burden of depression among patients with Type 2 DM treated in the private sector or secondary care. Also, we did not collect data on substance use including alcohol, family history of depression, availability of social support or recent stressors which might have had affected the results. A future study highlighting these variables should be conducted in order to plan a comprehensive patient-centered care plan. Despite these limitations, our study included a representative sample of the diabetic population that is managed in primary health care including older ages, different nationalities and sociodemographic backgrounds. Patients were recruited prospectively and were assessed for multiple potentially confounding variables in order to reduce selection bias. Also, the diagnosis of depression was based on the PHQ-9 assessment tool which has been validated previously for diagnosing depression in primary care; therefore, self-reported diagnosis of depression was avoided.

Based on the study results, patients with Type 2 DM should be regularly screened for depression, and those diagnosed with depression should be referred to social workers and/or psychologists if appropriate. Managing depression in diabetes with a multidisciplinary approach would facilitate patient-centered care and optimize control of their interrelated risk factors.

## 6 Conclusion

Depression is prevalent comorbidity among people with diabetes who are treated in primary health care. The current study showed that a fifth of the studied population was depressed with male patients being at a higher risk of developing depression while being Qatari and treatment of diabetes with insulin were associated with a lower risk for developing comorbid depression in people with diabetes.

## Ethical approval

Ethical approval was obtained from IRB from PHCC research department.

## Consent

Verbal informed consent was obtained from all participants.

## Authors contributions

All authors contributed to designing the research proposal, data collection, data analysis, writing, and all authors approved the final Manuscript.

## Funding

The authors have not declared a specific grant for this research from any funding agency in the public, commercial or not-for-profit sectors.

## Data availability

All data relevant to the study are included in the article or uploaded as supplementary information

## Declaration of Competing Interest

The authors declare that they have no known competing financial interests or personal relationships that could have appeared to influence the work reported in this paper.

## References

[1] Zhang P, Zhang X, Brown J (2010 Mar 1). Global healthcare expenditure on diabetes for 2010 and 2030. Diabetes research and clinical practice.

[2] Kalra S, Jena BN, Yeravdekar R. (2018 Sep). Emotional and psychological needs of people with diabetes. Indian journal of endocrinology and metabolism..

[3] World Health Organization (WHO) (2021). https://www.who.int/news-room/fact-sheets/detail/depression.

[4] Moussavi S, Chatterji S, Verdes E, Tandon A, Patel V, Ustun B. (2007 Sep 8). Depression, chronic diseases, and decrements in health: results from the World Health Surveys. The Lancet.

[5] Katon WJ. (2008 Nov 1). The comorbidity of diabetes mellitus and depression. The American Journal of Medicine.

[6] Egede LE, Zheng D, Simpson K. (2002 Mar 1). Comorbid depression is associated with increased health care use and expenditures in individuals with diabetes. Diabetes care.

[7] Vos T, Barber RM, Bell B, Bertozzi-Villa A, Biryukov S, Bolliger I, Charlson F, Davis A, Degenhardt L, Dicker D, Duan L. (2015 Aug 22). Global, regional, and national incidence, prevalence, and years lived with disability for 301 acute and chronic diseases and injuries in 188 countries, 1990–2013: a systematic analysis for the Global Burden of Disease Study 2013. The lancet.

[8] Champaneri S, Wand GS, Malhotra SS, Casagrande SS, Golden SH. (2010 Dec 1). Biological basis of depression in adults with diabetes. Current diabetes reports.

[9] Niraula K, Kohrt BA, Flora MS (2013 Dec). Prevalence of depression and associated risk factors among persons with type-2 diabetes mellitus without a prior psychiatric history: a cross-sectional study in clinical settings in urban Nepal. BMC psychiatry.

[10] Bener A, OAA Al-Hamaq A, E Dafeeah E (2011 Dec 9). High prevalence of depression, anxiety and stress symptoms among diabetes mellitus patients. The Open Psychiatry Journal..

[11] Egede LE, Ellis C. (2010 Mar 1). Diabetes and depression: global perspectives. Diabetes research and clinical practice.

[12] Ciechanowski P. (2011 Apr 1). Diapression: an integrated model for understanding the experience of individuals with co-occurring diabetes and depression. Clinical Diabetes..

[13] Lloyd CE, Roy T, Nouwen A, Chauhan AM. (2012 Oct 1). Epidemiology of depression in diabetes: international and cross-cultural issues. Journal of Affective Disorders.

[14] Wu SF, Huang YC, Liang SY, Wang TJ, Lee MC, Tung HH. (2011 Nov 1). Relationships among depression, anxiety, self-care behaviour and diabetes education difficulties in patients with type-2 diabetes: a cross-sectional questionnaire survey. International Journal of Nursing Studies.

[15] Kaur G, Tee GH, Ariaratnam S, Krishnapillai AS, China K. (2013 Dec). Depression, anxiety and stress symptoms among diabetics in Malaysia: a cross sectional study in an urban primary care setting. BMC family practice..

[16] Dejenie Habtewold T, Radie YT, Sharew NT (2015 Oct).

[17] Zahid N, Asghar S, Claussen B, Hussain A. (2008 Jan 1). Depression and diabetes in a rural community in Pakistan. . *Diabetes research and clinical practice*.

[18] Papelbaum M, Moreira RO, Coutinho W, Kupfer R, Zagury L, Freitas S, Appolinário JC. (2011 Dec). Depression, glycemic control and type 2 diabetes. Diabetology & metabolic syndrome.

[19] Al-Amer RM, Sobeh MM, Zayed AA, HA Al-Domi (2011 Jul 1). Depression among adults with diabetes in Jordan: risk factors and relationship to blood sugar control. Journal of Diabetes and its Complications.

[20] Tran NM, Nguyen QN, Vo TH, Le TT, Ngo NH. (2021). Depression Among Patients with Type 2 Diabetes Mellitus: Prevalence and Associated Factors in Hue City, Vietnam. Diabetes, Metabolic Syndrome and Obesity. Targets and Therapy.

[21] Mathew CS, Dominic M, Isaac R, Jacob JJ. (2012 Sep). Prevalence of depression in consecutive patients with type 2 diabetes mellitus of 5-year duration and its impact on glycemic control. Indian journal of endocrinology and metabolism.

[22] Sweileh WM, Abu-Hadeed HM, Al-Jabi SW, Sa'ed HZ (2014 Dec). Prevalence of depression among people with type 2 diabetes mellitus: a cross sectional study in Palestine. BMC public health.

[23] NA AM (2018). Prevalence of depression among type 2 diabetic patients attending diabetic clinic at primary health care centers in Jeddah, Saudi Arabia. Arch Med.

[24] Kroenke K, Spitzer RL. (2002). The PHQ-9: a new depression diagnostic and severity measure. Psychiatric Annals.

[25] Cameron IM, Crawford JR, Lawton K (2008 Jan). Psychometric comparison of PHQ-9 and HADS for measuring depression severity in primary care. Br J Gen Pract.

[26] Gelaye B, Williams MA, Lemma S, Deyessa N, Bahretibeb Y, Shibre T, Wondimagegn D, Lemenhe A, Fann JR, Vander Stoep A, Zhou XH (2013 Dec 15). Validity of the patient health questionnaire-9 for depression screening and diagnosis in East Africa. Psychiatry research.

[27] Khaledi M, Haghighatdoost F, Feizi A, Aminorroaya A. (2019 Jun). The prevalence of comorbid depression in patients with type 2 diabetes: an updated systematic review and meta-analysis on huge number of observational studies. Acta diabetologica.

[28] Bawadi H, Al-Shahwani A, Arafeh D, Al-Asmar D, Moawad J, Shi Z, Daher-Nashif S. (2021 Mar).

[29] Asefa A, Zewudie A, Henok A, Mamo Y, Nigussie T. (2020 Apr 12). Depression and its associated factors among diabetes mellitus patients attending selected hospitals in Southwest Ethiopia: a cross-sectional study. Psychiatry journal.

[30] Deischinger C, Dervic E, Leutner M (2020 Sep 1). Diabetes mellitus is associated with a higher risk for major depressive disorder in women than in men. BMJ Open Diabetes Research and Care.

[31] Arambewela MH, Somasundaram NP, Jayasekara HB, Kumbukage MP. (2019 Feb 3). Prevalence of depression and associated factors among patients with type 2 diabetes attending the diabetic clinic at a tertiary care hospital in Sri Lanka: a descriptive study. Psychiatry Journal.

[32] World population review, 2022. Available at https://worldpopulationreview.com/countries/qatar-population [accessed on February 14th 2022].

[33] Sunny AK, Khanal VK, Sah RB, Ghimire A. (2019 Jun 10). Depression among people living with type 2 diabetes in an urbanizing community of Nepal. Plos one.

[34] Abou Abbas L, Salameh P, Nasser W, Nasser Z, Godin I. (2015 Feb). Obesity and symptoms of depression among adults in selected countries of the Middle East: a systematic review and meta-analysis. Clinical obesity..

[35] Richardson LK, Egede LE, Mueller M, Echols CL, Gebregziabher M. (2008 Nov 1). Longitudinal effects of depression on glycemic control in veterans with Type 2 diabetes. General hospital psychiatry.

[36] Lustman PJ, Clouse RE. (2005 Mar 1). Depression in diabetic patients: the relationship between mood and glycemic control. Journal of Diabetes and its Complications.

[37] Ismail K, Winkley K, Rabe-Hesketh S. (2004 May 15). Systematic review and meta-analysis of randomised controlled trials of psychological interventions to improve glycaemic control in patients with type 2 diabetes. The Lancet.

